# Evaluation of Functionality and Biological Responses of *Mytilus galloprovincialis* after Exposure to Quaternium-15 (Methenamine 3-Chloroallylochloride)

**DOI:** 10.3390/molecules21020144

**Published:** 2016-01-26

**Authors:** Maria Pagano, Gioele Capillo, Marilena Sanfilippo, Simon Palato, Francesca Trischitta, Antonio Manganaro, Caterina Faggio

**Affiliations:** Department of Chemical, Biological, Pharmaceutical, and Environmental Sciences, University of Messina, Viale F. Stagno d’Alcontres, 31, 98166 Messina, Italy; mariapagano88@gmail.com (M.P.); gcapillo@unime.it (G.C.); msanfilippo@unime.it (M.S.); simon.palato89@gmail.com (S.P.); ftrischi@unime.it (F.T.); amanganaro@unime.it (A.M.)

**Keywords:** *Mytilus galloprovincialis*, quaternium-15, gill histopathology, regulatory volume decrease, citotoxicology

## Abstract

Although the irritant effects of quaternium-15 have been established, little is known about the toxicological consequences induced by this xenobiotic on aquatic invertebrates. The present article reports toxicological, histological and physiological effects of quaternium-15 following the exposure of *Mytilus galloprovincialis* for 18 days at three different concentrations (0.1, 1.0 and 2.0 mg/L). The results demonstrate that at higher concentrations histological damages to *M. galloprovincialis* gills occur, like melanosis, light exfoliations, increase of mucus production and infiltrative inflammation. In addition digestive gland cells of *M. galloprovincialis*, were not able to perform the regulation volume decrease (RVD) owing to osmotic stress following the exposure to the preservative. Overall, this first study on quaternium-15 highlights that it can jeopardize both the morphology and vital physiological processes in marine invertebrates, depending on the duration of exposure and the concentration of the preservative, indicating that further studies are necessary to increase our knowledge about the effects of this substance, commonly added to our products of daily use.

## 1. Introduction

Nowadays the use of preservatives is an important part of anthropogenic activities and from the second half of the twentieth century on there has been an exponential growth in their consumption. A preservative is a natural or synthetic ingredient that is added to products such as foods, pharmaceuticals and personal care products to prevent spoilage, whether from microbial growth or undesirable chemical changes. This situation has led to a progressively increasing discharge of large amounts of these products, which, via sewage, then reach the aquatic environment, affecting the organisms living there. The presence of preservative residues in the aquatic environment has recently received great attention, since high levels of contamination have been found in open waters [1,2]. Moreover, these compounds are specifically designed to resist metabolic degradation, and high lipophylicity is generally a basic requirement to maximize absorption by target organisms [3]. In Europe, the list of preservatives allowed in cosmetic products is over 50. Among these substances hexamethylenetetramine chloroallyl chloride (quaternium-15) is a quaternary ammonium salt widely used as a surfactant and preservative in many cosmetics and industrial substances. Quaternium-15 at 0.1% concentration (1000 ppm) releases 100 ppm of free formaldehyde; this amount is enough to induce dermatitis in a patient with formaldehyde sensitivity [4,5,6]. The degree of completeness of formaldehyde release in a cosmetic product will depend on several factors, such as the concentration of the preservative in the product, the percentage of water in the product, the rate of formaldehyde release from the specific preservative, and the length of time since formulation. Sources of quaternium-15 are shampoos, hair conditioners, liquid soaps, shaving products, moisturizing creams and lotions, cosmetics, and sunscreens, topical medications, cleansers, disinfectants, and laundry soaps, gloves, metalworking fluids and cutting fluids, latex paints, glues, and adhesives; food packaging: paper, paperboard, and polyurethane resins [7,8,9,10].

Quaternium-15 has already been the subject of various scientific studies regarding its irritant effects [11,12]. No data exist however about its effect on aquatic animals. Different tools have been used to compare responses of marine organisms in closely related populations to multiple environmental stresses in order to better understand cellular processes involved in the adaptive mechanisms. Thus, the purpose of this work is to understand the impact of exposure the quaternium-15 on *Mytilus galloprovincialis* digestive gland osmotic stress response and possible morphological alterations of gills and digestive gland. This invertebrate sessile bivalve, being directly exposed to abiotic and contamination stresses, constitutes a good sentinel species for environmental studies, as already shown by other authors [13,14,15,16]. The survival of organisms coping with an additional stress (xenobiotic exposure) could jeopardize their ability of adaptation and compromise population maintenance. Owing to their filter feeding ability they bioaccumulate most chemicals associated with fine particles. Mussels, of the genus *Mytilus*, are one of the most common marine molluscs, and thus, they are significant components of coastal ecosystems and important for human food consumption. Thus, mussels reflect local contamination history by integrating the environmental exposure. Furthermore, *M. galloprovincialis* is a convenient and adequate model in toxicological studies because the species is easy to maintain and test under laboratory conditions [16,17,18,19,20]. These organisms represent an important model for physiological studies, as being osmoconformers, they can resist large fluctuations of environmental parameters by triggering efficient adaptive mechanisms. Among the mechanisms of adaptation, Regulatory Volume Decrease (RVD), represents an integrative process that most animal cells, also in mussels, perform to counteract hypoosmotic swelling. This regulatory response is accomplished through the loss of intracellular solutes along with osmotically obligated water. The maintenance of cell volume and its restoration after the “disturbance” following the exposure to xenobiotics, allows cells to protect themselves from lysis [21]. In view of the limited knowledge of the effects of quaternium-15 on marine molluscs, the present work aims at determining the effects of quaternium-15 in *M. galloprovincialis* using the RVD effect and histological appraisals. To our knowledge, this is the first time that the quaternium-15 effect has been tested both on osmotic stress index and on morphologic alterations in marine invertebrates.

## 2. Results

### 2.1. Monitoring Parameters

During the experimental period, temperature, salinity and pH have been constantly monitored, also before and after the change of water, to ensure the maintenance of suitable environmental conditions for the life of the mussels, as close as possible to those of the natural environment.

### 2.2. Histopathological Condition Indices

For histopathological condition indices five histological changes were considered for the weighted indices approach. Table 1 summarizes the classifications of histopathological changes, derived by the following formula:

$$I_{h}\;=\;\frac{\sum_{1}^{j}w_{j}a_{\text{jh}}}{\sum_{1}^{j}M_{j}}$$

**Table 1:** Average gill histopathological condition indices (±95% confidence intervals) for each tank and respective alteration weights.

Histopathological Parameters	Alteration	w	1.0 mg/L	2.0 mg/L
Cellular and morphological changes			0.48 ± 0.04	0.74 ± 0.06
	Melanin/lipofuscin deposit	1		
	Loss of cilia	2		
	Enlarged central vessel	1		
	Haemocyte infiltration	1		
	Hypertrophy of goblet cells (GC)	2		

w = alteration weights. The values are means ± SE. *p* < 0.05 respect to the control condition (*t*-test).

The histopathological condition indices of the gills of specimens from tanks at different concentrations of quaternium-15, reveal that there is a significant difference between the two higher exposure groups, derived from the weight attributed to the hypertrophy of goblet cells (GC) morphological change. The animals exposed to 0.1 mg/L of quaternium-15 did not show differences compared to control animals (*i.e*., histopathological condition indices were absent).

### 2.3. Histopathology

**Figure 1:**
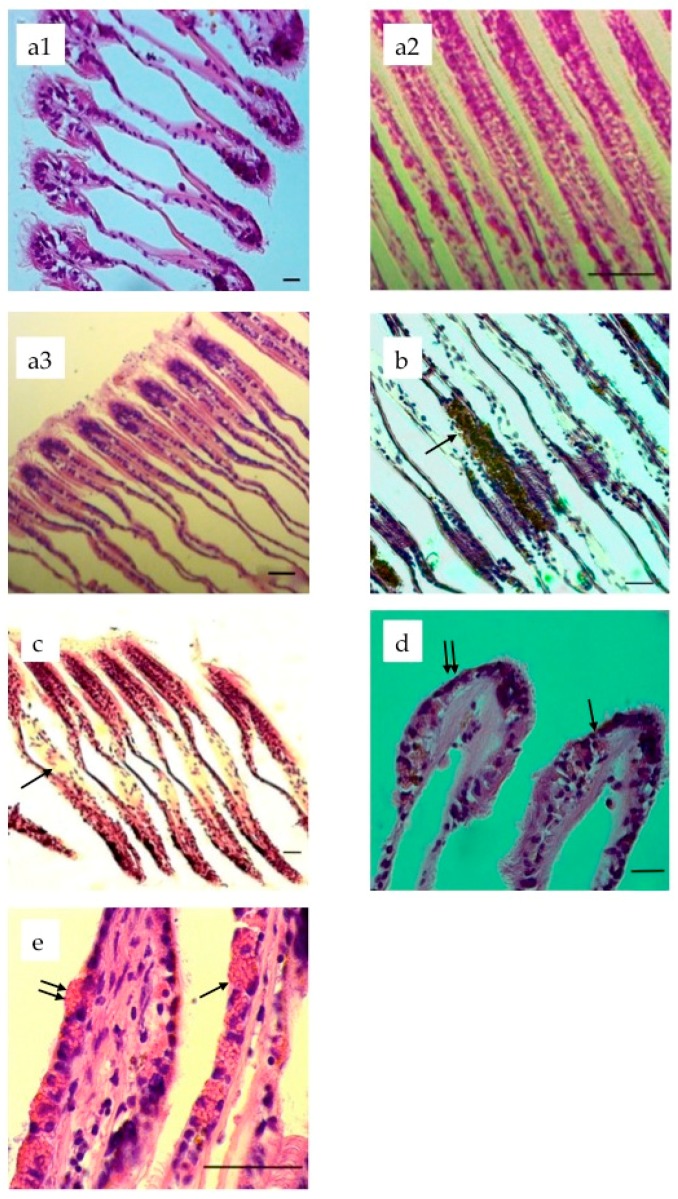
Representative histological sections of *Mytilus galloprovincialis* gills (H & E staining), normal gills of a control mussel 40× (**a1**), 20× (**a2**) and 10× (**a3**); animals exposed to 1.0 mg/L of quaternium-15, after the 18-day exposure, melanin deposit (arrow) and lack of cilia (single arrow), 20× (**b**); animals exposed to 1.0 mg/L of quaternium-15 after the 18-day exposure, gill filaments filled with haemocytes (arrow), 10× (**c**); animals exposed to 2.0 mg/L of quaternium-15, after the 18-day exposure, haemocytes inside the epithelium (arrow), and lack of cilia (double arrows), 63× (**d**); animals exposed to 2.0 mg/L of quaternium-15, after the 18-day exposure, goblet cells increased in volume with granules inside cytoplasm (single arrow). Double arrows indicate these granules probably during releasing process, 100× (**e**). Each scale bar = 50 μm.

Histopathological observations did not show significant lesions in the digestive glands of any of the specimens from the different concentration tanks. Compared to the specimens from the control tank (Figure 1a), no acute responses in the gills of mussels were found in the tank with the concentration of 0.1 mg/L. Instead in gills of *Mytilus* spp. from the tanks with concentration of 1.0 and 2.0 mg/L, different damage and acute responses were present after 18 days of exposure, with no marked differences between the two tanks. The first reaction of the tissue was the massive deposit of melanin (dark brown staining) in the gill epithelium (Figure 1b). Frontal and lateral cilia were often missing (Figure 1b), with enlarged central vessels of filaments filled with haemocytes (Figure 1c). In a different case haemocytes were found in the branchial epithelium outside the lumen (Figure 1d), probably due to diapedesis, caused by inflammation (see Discussion). The only difference in the gill tissue responses at the different concentrations (1.0 and 2.0 mg/L) was that, in specimens of the tank at the higher concentration, the size of goblet cells was increased compared to the mussels in the 1.0 mg/L concentration tank. In addition to this, and as a result of the hypertrophy of mucous cells, there was overproduction of mucus stored in granules at the apex of the goblet cells, with respect to the control specimens. In some cases it was possible to see these granules released out of the tissue (Figure 1e). The massive production of mucus by gills and other tissues caused the sliminess of the specimens noted during collection from the tanks. In fact there was an increased sliminess of the specimens of the tank with 2.0 mg/L of quaternium-15 compared with the lower concentration tanks (Figure S1).

### 2.4. RVD Experiments in Digestive Cells

In order to evaluate the response to hypotonic solution, isolated digestive cells from animals in the control group have been exposed to a rapid change of osmolarity (1100–1800 mOsm/kg). One drop of cell suspension was placed on a glass slide pretreated with poly-lysine to facilitate cell adhesion. Two thin strips of double-sided adhesive were placed at the upper and lower edges of the glass slide to support the cover slip and to create an interspace in which the experimental solutions were added. They were placed at one side of the cover slip with a pipette and were absorbed at the opposite side with strips of filter papers. This allowed a rapid change (a few second) of the solution in the interspace. Cells were observed with a light microscope (Leitz Diaplan, Wetzlar, Germany); the videometric measurements were carried out on the digestive cells, the most abundant type in all preparations. Cell images were digitized using a colour video camera (Sony, Tokyo, Japan) that was connected to a PC. Individual cells were selected; images were taken a various time intervals, as described in the succeeding text, and recorded on PC. In iso-osmotic control tests, the images were taken at 0 and 3 min in isotonic solution; afterwards, the solution was rapidly changed with an identical solution, and the images were taken every 1 min for the first 10 min after the change of the solution and thereafter every 5 min for 20 min.

**Figure 2:**
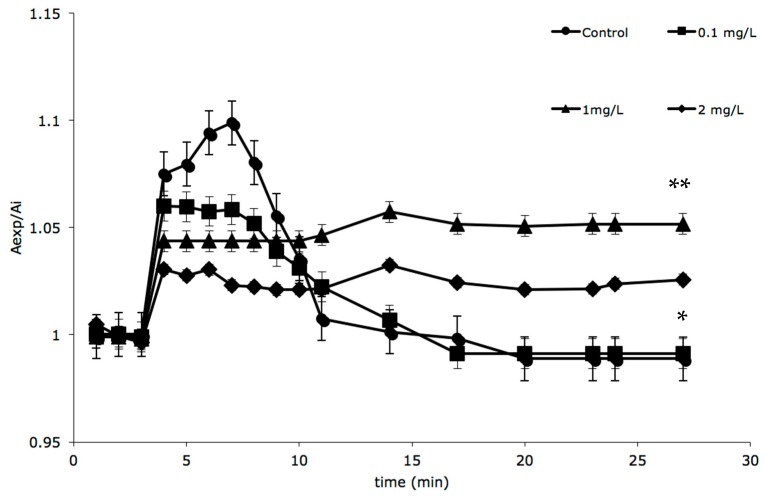
Relative area changes of cells isolated by digestive gland of *M. galloprovincialis* exposed to hypotonic solution, after 18 days of exposure to quaternium-15: control (●), 0.1 mg/L (■); 1.0 mg/L (**▲**); 2.0 mg/L (♦); number of animals = 8. The values are means ± SE. * *p* < 0.05 respect to the maximum swelling; ** *p* < 0.05 respect to the control condition (two way ANOVA test).

The cells exposed to the rapid change of osmolarity initially increased in size and then tended to return to their initial volume. As Figure 2 shows, the cells reached their maximum swelling, corresponding to an 11% increase in volume, after 1 min of exposure to the hypotonic medium. Thereafter, they exhibited an RVD response. The digestive cells isolated from animals, exposed to quaternium-15 (0.1 mg/L) for 18 days, exhibited an RVD response as much as control cells. In contrast, digestive cells, isolated from animals exposed to higher quaternium-15 concentrations (1.0 mg/L and 2.0 mg/L) for 18 days, were not able to perform RVD.

## 3. Discussion

This study shows that quaternium-15 produces both physiological and morphological alterations in *M. galloprovincialis.* The observation that the main water parameters (temperature, salinity and pH) were unchanged in all tanks throughout the experimental period rules out the possibility that the observed alterations were due to deteriorated environmental conditions.

In fact, temperature strongly influences the respiration rates (direct relation), the excretion rates (above 25 °C) and salinity is proved to have a strong influence on clearance rates, which are inhibited at low salinities [22,23]. Results of other studies of pH effects in bivalve molluscs showed that some deleterious effects set in at pH values around 7.5, and all studies show that marine bivalve molluscs cannot tolerate pH values ≤ 7.0 [24,25,26,27].

To date, an extensive bibliography exists on quaternium-15 regarding its irritant effects on mammals [28,29,30,31,32,33,34,35]. Being a preservative added to various pharmaceutical, personal care products and intensely used, as the last step of its environmental fate it reaches the water environment. Despite this fact, there is no information on its effects on marine species in general. For this reason, the effect of this substance at three different concentrations has been tested on *M. galloprovincialis*. Exposure of bivalve molluscs to different xenobiotics (chemical additive in personal care products, drugs) and heavy metals has been studied by many authors [3,14,36,37,38,39,40,41,42,43,44,45,46]. In some of these papers, morphological and histological studies have been carried out on different tissues. Marine bivalve molluscs, and in particular *Mytilus* species, are widely used as sentinel organisms and represent a relevant model for studies of morphological and physiological regulatory responses, as they can resist large fluctuations of environmental parameters by triggering efficient adaptive mechanisms [14,47]. These excellent adaptive and defence mechanisms well explain the resistance of our specimens exposed to the lower concentration of the toxicant (0.1 mg/L) during experimentation.

After 18 days of exposure to higher concentrations of the xenobiotic (1.0–2.0 mg/L) different damage and alterations of branchial tissue were present (see Results). Between the two higher concentrations (1.0–2.0 mg/L) there are no relevant differences, so this demonstrates that the maximum level of resistance and adaptation of the organism to exposure has been exceeded, as already reported [39].

The massive deposit of melanin, in tissues that normally contain it, is known as melanosis [48]. Melanosis demonstrates an inflammation of the tissue as result of the “pro-phenoloxidase (PO) activating systems” that play a basic role in immune mechanisms involved in the phenomena of recognition, cytotoxicity and encapsulation of foreign bodies. This activity has been reported in different bivalve molluscs [49].

The lack of cilia has been already reported [39], and it should be considered as the beginning of gill exfoliation, damage already observed in clams and, with less incidence, in *M. galloprovincialis* [38]. They indicate that the severe exfoliation of the gill filaments will lead, in the short term, to difficulties in filtering food, and in the mid-term, to breathing problems, compromising the survival of the animals in a serious way.

The central vessel filled with haemocytes derives from an increased flow of hemolymph, compared to control specimens, that brings defensive cells to the site of inflammation, in fact in some cases haemocytes were in a diapedesis process and inside the epithelium too. This process allows phagocytosis. In our case, the type of inflammation is infiltrative. Haemocyte infiltration in bivalve molluscs gills is often observed in specimens exposed to xenobiotics as a defence reaction to cellular damage induced by multiple environmental contaminants. The gills are organs particularly subjected to the accumulation of toxic molecules because they are involved in the respiration and feeding processes, and they are also of peculiar interest because they represent a barrier between the whole body of the mussel and the outside aquatic environment [50,51].

The change in morphology of goblet cells (GC) shows a relative increase of the tissue exposure to the toxicant. Granules at the apex of the GC, reversed outside the branchial epithelium demonstrate the reaction of the tissue to inflammation, and this has been already reported, in less intense phenomena [40]. This reaction should depend on a particular mechanism of defence called MXDM in bivalve molluscs, discovered in a freshwater bivalve mollusc, *Anodonta cygnea*. MXDM can protect cells from toxic compounds by limiting entry and in facilitating efflux of these compounds [52].

There were no significant lesions on digestive glands. The absence of significant lesions on digestive gland, also reported by other authors [37], should result from the fact that the digestive gland of mussels is the analogue of the vertebrate liver and therefore, the primary organ for detoxification. However an alteration of the organ functionality is shown by the RVD experiments.

It has recently become clear that a reduction in cell volume can also serve as specific signal in the regulation of physiological processes, so it is evident that there is a relationship between cell volume regulation and organism physiology/pathophysiology [53,54,55]. As show in Figure 2, under control conditions (without quaternium-15), the isolated digestive cells of *M. galloprovincialis* exhibited a partial RVD and similar results have been obtained in a previous study [21,47]. The digestive cells of animals exposed to the preservative (1.0 and 2.0 mg/L quaternium-15) were unable to perform RVD, while in cells of animals exposed to lower concentration (0.1 mg/L quaternium-15) RVD was not affected (Figure 2 and Figure S2). However the initial swelling has not been jeopardized when the hypotonic shock has been applied in cells isolated from the digestive glands of animals exposed for 18 days to any concentration of quaternium-15. The impairment of the homeostatic response has been observed in two experimental conditions (exposure to 1.0 and 2.0 mg/L), even if the digestive cells maintained their viability. In fact, the lysosome stability, evaluated by the neutral red retention assay, has been not altered compared with control values (data not shown).

The lower concentration doesn’t compromise RVD, probably because the cell is able to cope with low concentrations of quaternium-15 but not with higher concentrations. An increase in dose may raise the intensity of an effect, or a more severe effect may result. The observation is that the quaternium-15 was ineffective on the osmotic water permeability while decreasing the swelling activated by solute permeability. The initial swelling, due to the entry of water following the osmotic gradient, was about 11% and this was identical either in the control or in quaternium-15 treated cells (Figure 2). Quaternium-15 could interact with lipid membranes, disrupting membrane integrity and causing toxic effects, as another researcher has demonstrated for other lipophilic substances [56]. The plasma membrane of the cell is a serious and important barrier, and the lack of integrity is probably caused by interference with membrane permeability or membrane proteins. In the light of these observations it is conceivable that the impairment of RVD in cells of quaternium-15 exposed animals is due to the lack of membrane integrity that does not allow the activation and/or the insertion of membrane transporters involved in ion movement from the cells, leading to osmotic water efflux during RVD (Figures S3 and S4).

## 4. Experimental Section

### 4.1. Animal Collection

The Mediterranean mussels *M. galloprovincialis* Lamarck, 1819 (Mollusca, Bivalvia) have been obtained from a mariculture facility (Company SACOM, Faro Lake, Messina, Italy) and transferred in 10 min to a tank containing synthetic natural sea water (SNSW, Nutri-SeaWater^®^Aquarium Saltwater, (pH = 8 ± 0.1; salinity= 37 ± 1 T = 17 °C ± 1 °C; 1100 mOsm/kg) with continuous aeration. After 1 week of acclimation, mussels have been randomly divided into four tanks (20 liters) with 35 animals each, filled with the same SNSW and kept under the conditions described above. One control tank and three tanks have been subjected to different concentrations of quaternium-15: 0.1 mg/L; 1 mg/L and 2 mg/L. The mussels were fed once a day with algal slurry (Liquifry Marine, Interpet, Dorking, UK) and SNSW has been changed every two days. Whenever water was changed, quaternium-15 concentrations were reestablished. The exposure lasted for 18 days. No mortality has been registered during the exposure. Hexamethylenetetramine chloroallyl chloride, 96% (Quaternium-15) has been obtained from Alfa Aesar GmbH & Co. KG (Karlsruhe, Germany).

### 4.2. Water Monitoring

During the experimental period, temperature, pH and salinity have been constantly monitored in all the tanks to assess the quality of the waters and to evaluate the possible influence of environmental parameters on the physiology of organisms [23,49]. All parameters have been measured with a multiparametric probe YSI 85 System (YSI Incorporated,Yellow Spring, OH, USA) and a HI 83140 pH-meter (HANNA Instrument, Padova, Italy).

### 4.3. Histological Analysis

Three specimens of *M. galloprovincialis* have been collected from each tank after 18 days of exposure to the quaternium-15. Fresh tissues (gills and digestive glands, Figure S5) have been fixed in immunofix (paraformaldheyde 4% in phosphate saline buffer, Bio-Optica, Milan, Italy) for 8 h at room temperature. After dehydration through an ascending ethanol series, the tissues have been infiltrated with xylene before embedding in paraffin. Serial sections, 5 μm thick, have been carried out from the paraffin block by a RM2235 manual rotary microtome (Leica, Buffalo Grove, IL, USA). The sections have been stained with hematoxylin (Harris’) and counterstained with eosin (H & E staining), cleared with xylene and mounted with Eukitt (Bio-Optica). A qualitative histopatological screening has been performed focusing on digestive glands and gills, under a light microscope (Leitz Diaplan), using different objectives 10×, 20×, 40×, 63×, 100× in oil immersion.

### 4.4. Histopathological Condition Indices

Semi-quantitative histopathological indices (*I_h_*) have been estimated for each individual based on the weighted indices approach proposed by Bernet *et al.* [57] for fish, with modifications according to Costa *et al.* [58]. At each alteration is attributed a weight (biological significance) with value ranging between 1 and 3 (maximum severity), and a score (degree of dissemination) with values between 0 (in the case that alteration is not present) and 6 (in the case alteration is diffuse). The formula for the estimation of histopathological condition indices is:

$$I_{h}\;=\;\frac{\sum_{1}^{j}w_{j}a_{\text{jh}}}{\sum_{1}^{j}M_{j}}$$

where *I*_h_ is the histopathological condition index for the individual *h*; *w*_j_ the weight of the *j*th histopathological alteration; *a*_jh_ the score attributed to the *h*th individual for the *j*th alteration and *M*_j_ is the maximum attributable value for the *j*th alteration, *i.e.*, weight × maximum score. The equation’s denominator normalizes *I*_h_ to a value between 0 and 1, thus permitting comparisons between distinct situations (such as different organs). The indices have been globally estimated per organ (gills) and subdivided by “reaction patterns”: cellular and morphological epithelial changes (gills).

The condition weights proposed have been based on observations carried out in this experiment and partially on literature concerning both invertebrate [58] and vertebrate histopathology [57,59,60]. In accordance, goblet cells hypertrophy and loss of cilia (w = 2). Intracellular melanin/lipofuscin-like deposits have been given the lowest weight (w = 1), as for inflammation-related alterations (e.g., haemocytic infiltration), and enlarged central vessel.

### 4.5. Isolation of Digestive Cells and RVD Experiments

The RVD experiment has been done after 18 days. For the experiment, four pools, each one composed by the digestive glands of five animals have been isolated according to the method described by Dailianis and Kaloyianni [61] in order to obtain an adequate number of cells. Digestive glands have been cut in pieces and washed with a Ca^2+^ and Mg^2+^ free solution (Sol. 1: NaCl 600 mM; KCl 12.5 mM; HEPES 20 mM), transferred to a test tube containing 0.01% collagenase (type IV-activity P125 CDU/mg; CDU = collagenase digestion units, Sigma–Aldrich, St. Louis, MO, USA) and dissolved in Sol. 1. The test tube has been gently stirred for 60 min at 18 °C in a thermostatic bath. Afterwards the suspension has been filtered through 200 μm and 75 μm nylon filters. The cells have been suspended in physiological saline solution (Sol. 2: NaCl 550 mM; KCl 12.5 mM; MgSO_4_ 8 mM; CaCl_2_ 4 mM; glucose 10 mM, HEPES 20 mM), washed twice by centrifugation (500 rpm/10 min/4 °C) and then resuspended in Sol. 2. Before the experiments the cells have been maintained in physiological saline solution (Sol. 2) at 18 °C for 1 h to re-establish ionic concentration on either side of the cell membrane.

For the RVD experiments the isolated cells have been visualized and measured by the method described in a previous paper [21]. One drop of cell suspension has been placed on a glass slide pretreated with poly-lysine to facilitate cell adhesion.

Two thin strips of double-sided adhesive have been placed at the upper and lower edges of the glass slide to support the cover slip and to create an interspace in which the hypotonic experimental solution has been added later (Sol. 3: NaCl 350 mM; KCl 12.5 mM; MgSO_4_ 8 mM; CaCl_2_ 4 mM; glucose 10 mM, HEPES 20 mM). The experimental solutions have been placed at one side of the cover slip with a pipette and were absorbed at the opposite side with strips of filter papers. This allowed a rapid change (in a few seconds) of the solution in the interspace. Cells were observed with a light microscope (Leitz Diaplan) and video-metric measurements have been carried out on digestive cells. Cell images have been digitized using a color video camera (Sony) connected to a PC. Individual cells have been selected; images have been taken at various time intervals and recorded on PC. The profile of the cells has been drawn with the aid of ImageJ (NIH, Bethesda, MD, USA). The cell areas for each experimental condition (Aexp) have been compared to the areas measured in isotonic solution (Ai) at the beginning of the experiment. Consequently the data are reported as relative area Aexp/Ai.

### 4.6. Statistical Analysis

One-way ANOVA has been used to test the differences between control and treatment and the Tukey test allowed pairwise comparisons among experimental conditions (*p* < 0.05). For histological experiments the *t*-test has been used. Results are expressed as means ± SE. The value of *p* < 0.05 was accepted as significant. The software package Prism 5.00 (2003, Graphpad Software Inc., La Jolla, CA, USA) has been used for statistical analysis.

## 5. Conclusions

In summary, the histological alterations found in the gills and the impairment of RVD machinery in digestive cells of *M. galloprovincialis* represent evidence of the environmental quality of water and indicates that these mussels could suffer damage if chronically exposed to toxic substances in their living environment. The current study demonstrates the application of gill histopathology and the assessment of cell volume regulation as a tool for monitoring water quality within aquatic ecosystems. The considerable variation in levels of different water xenobiotics highlights the important role of ecological and physiological factors in concentrating pollutants. This is the first study demonstrating that quaternium-15 has damaging effects on marine organisms. The adverse effects recorded in the current study have not been severe enough to cause mussels’ mortality, but they might potentially lead to lethal effects if the mussels were exposed to higher concentrations of quaternium-15 over a longer period. Therefore, it is especially important that the application of such substances complies with measures to prevent their penetration into the aquatic environment. Our data illustrates the need for further research on the ecological consequences of xenobiotics in non-target organisms. The potential use of the results of this study in relation to public health, as *M. galloprovincialis* is important for human food consumption, will be explored in future studies.

## Supplementary Materials

Supplementary materials can be accessed at: http://www.mdpi.com/1420-3049/21/2/144/s1.
