# Supplementary material for: Evaluation of Functionality and Biological Responses of Mytilus galloprovincialis after Exposure to Quaternium-15 (Methenamine 3-Chloroallylochloride)
Source: Molecules. 2016 Jan 26;21(2):144. doi: 10.3390/molecules21020144 (PMC6273939; doi:10.3390/molecules21020144)
Supplement: Supplementary file 1 [file molecules-21-00144-s001.pdf]

Supplementary Materials: Evaluation of Functionality and Biological Responses of *Mytilus galloprovincialis* after Exposure to Quaternium-15 (Methenamine 3-Chloroallylochloride)

Maria Pagano †, Gioele Capillo †, Marilena Sanfilippo, Simon Palato, Francesca Trischitta, Antonio Manganaro and Caterina Faggio *

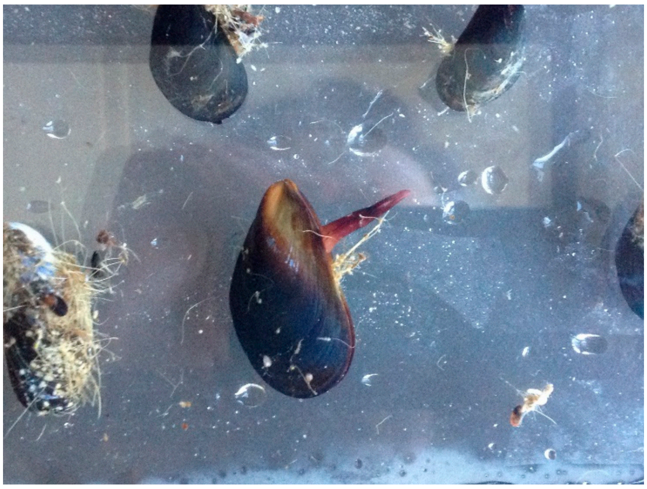

Figure S1. Evidence of mucus production in the tank in presence of quaternium-15 (2 mg/L).

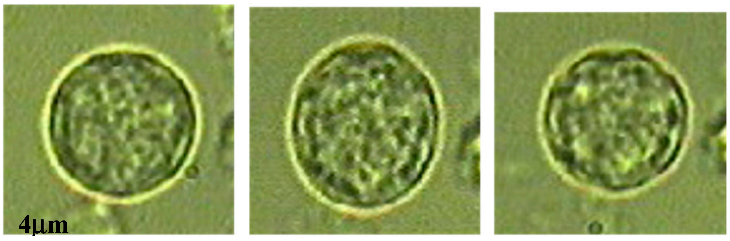

Isotonic Hypotonic (8min) Hypotonic (20 min)

Figure S2. Digitized images of a digestive cell showing area changes evoked by rapid shifts in the hypotonic solution. After the swelling the cell recovers own volume.

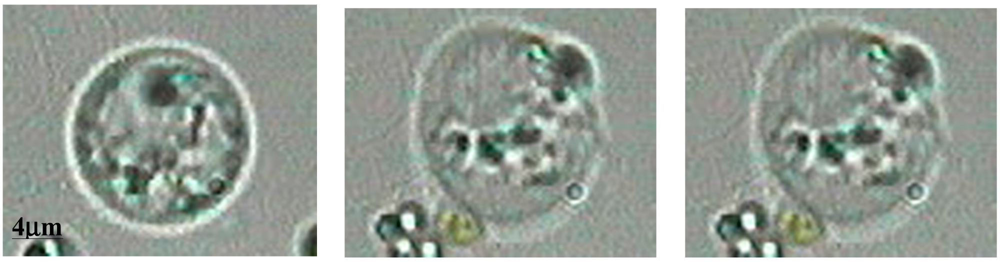

Isotonic Hypotonic (8min) Hypotonic (20 min)

Figure S3. Digitized images of a digestive cells, preincubated in quaternium-15 (1 mg/L), showing area changes evoked by rapid shifts in the hypotonic solution. After the swelling the cell doesn't recover own volume.

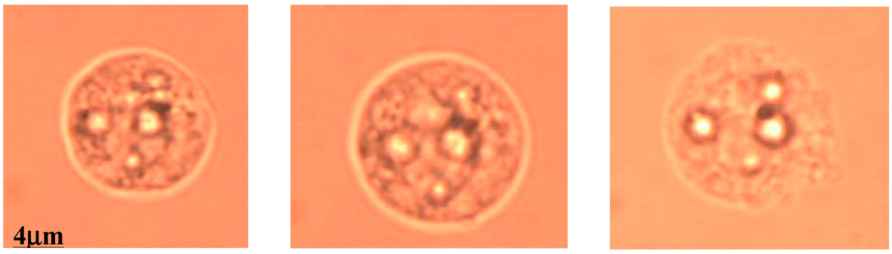

Figure S4. Digitized images of a digestive cells preincubated in quaternium-15 (2 mg/L) showing area changes evoked by rapid shifts in the hypotonic solution. After the swelling the cell doesn' t recover own volume.

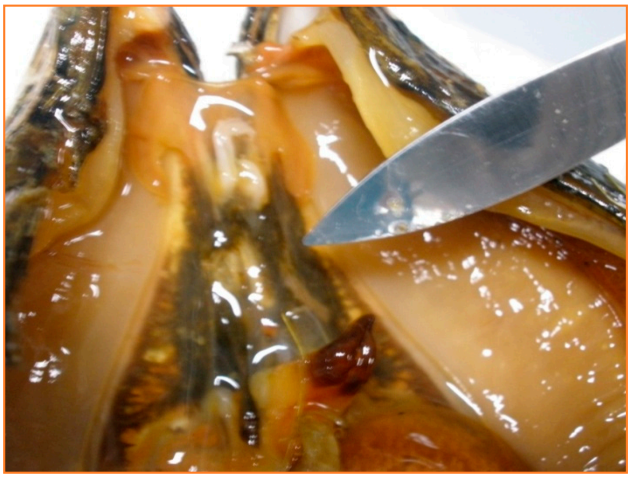

Figure S5. Hepatopancreas before the isolation.
